# Airway hydration homeostasis in artificial airways: from mucus biophysics to monitoring and humidification strategies in critical care: a narrative review

**DOI:** 10.3389/fmed.2026.1922639

**Published:** 2026-09-11

**Authors:** Haishao Chen, Xunbin Qiu, Kaisheng Ye, Qun Wang

**Affiliations:** 1 Department of Anesthesiology, Binhaiwan Central Hospital of Dongguan, Dongguan, Guangdong, China; 2 Department of Anesthesiology, The First Affiliated Hospital of Jinan University, Guangzhou, Guangdong Province, China

**Keywords:** airway humidification, airway surface liquid, artificial airway, biosensing, mechanical ventilation, mucociliary clearance, mucus rheology

## Abstract

Artificial airway placement bypasses the physiological warming and humidifying functions of the upper airway. This produces a distal shift of the isothermic saturation boundary and exposes the tracheobronchial mucosa to additional evaporative stress. When inspired gas is inadequately conditioned, the resulting evaporative stress can deplete airway surface liquid, concentrate mucus, impair mucociliary clearance, and promote secretion retention and mucus plugging. Airway hydration imbalance is not simply a problem of insufficient water delivery. Rather, it reflects a dynamic interaction among gas thermodynamics, epithelial ion transport, mucin network organization, inflammatory burden, secretion biophysics, and ventilator-patient conditions. Current humidification strategies, mainly heated humidifiers and heat-and-moisture exchangers, provide essential support during invasive and non-invasive ventilation. However, their performance is often constrained by open-loop operation, device-related limitations, and heterogeneous patient requirements. This review summarizes the physiological basis of airway hydration, the mechanisms by which artificial airways disturb hydration homeostasis, clinical assessment approaches, and current humidification strategies. It also discusses newer approaches, including ventilator waveform-derived indices, microrheological assays, artificial cilia sensors, microfluidic platforms, and closed-loop humidification frameworks. Future studies should determine whether integrating patient characteristics, secretion properties, ventilatory conditions, and device performance can support more individualized humidification strategies. Technologies that remain at the model or prototype stage should be interpreted cautiously.

## 1 Introduction

Artificial airways, including endotracheal tubes and tracheostomy tubes, are indispensable for securing the airway and delivering mechanical ventilation in critically ill patients. Their physiological cost is substantial: the upper airway, which normally warms, humidifies, and filters inspired gas, is partially or completely bypassed. If the inspired gas is inadequately conditioned, water and heat are extracted from the tracheobronchial mucosa, leading to airway surface liquid (ASL) depletion, mucus concentration, impaired mucociliary clearance (MCC), and secretion retention (1–4).

Humidification management has traditionally been discussed as a comparison between heated humidifiers (HHs) and heat-and-moisture exchangers (HMEs). This device-centered view remains clinically useful, but it does not fully capture the biological process that determines whether secretions remain transportable and removable. Airway hydration homeostasis is influenced by absolute humidity, temperature, ventilatory flow, secretion production, inflammatory burden, epithelial transport, and the mechanical environment created by the artificial airway.

Here, artificial-airway humidification is framed as a problem of maintaining airway hydration homeostasis rather than simply choosing between devices. The review synthesizes clinical guidelines, physiological studies, mucus biophysics, and monitoring technologies. Because the evidence comes from heterogeneous sources, including chronic airway disease models, *in vitro* microrheology, and early-stage engineering studies, mechanistic conclusions are interpreted according to their level of clinical validation.

## 2 Evidence framing and scope

### 2.1 Literature search and selection strategy

To support this narrative review, we conducted a structured literature search in PubMed and the Web of Science Core Collection, with the final search performed in May 2026. The search combined controlled vocabulary, where available, and free-text terms related to artificial airways, airway humidification and hydration, airway surface liquid, mucus biophysics and rheology, mucociliary clearance, heated humidifiers, heat-and-moisture exchangers, and emerging monitoring technologies. English-language clinical guidelines, randomized and non-randomized clinical studies, observational studies, physiological and disease-model investigations, and relevant engineering proof-of-concept studies were considered. Records retrieved from the two databases were deduplicated before screening. Haishao Chen, Xunbin Qiu, and Kaisheng Ye jointly screened titles and abstracts and reviewed potentially relevant full texts. Disagreements regarding eligibility were adjudicated by the corresponding author, Qun Wang. Because this was a narrative review, no formal risk-of-bias or certainty-of-evidence assessment was performed, and no PRISMA flow diagram was prepared. Database-specific search strategies and selection procedures are provided in Supplementary Table S1.

### 2.2 Evidence framing

The available literature spans several evidence layers. Direct clinical evidence supports the need for humidification during mechanical ventilation and provides practical targets for absolute humidity and temperature (1, 2). Mechanistic evidence concerning ASL regulation, periciliary layer compression, and mucin concentration is supported mainly by epithelial biology, cystic fibrosis, asthma, and mucus biophysics studies (4–12). Translational evidence for ventilator waveform analytics, *in situ* sensors, and closed-loop humidification is promising but remains preliminary (21–28).

Accordingly, the following sections separate established clinical recommendations from mechanistic extrapolations and early technological concepts. This framing helps avoid overstating causal links between airway dehydration, mucus biophysics, and clinical complications. These evidence layers are summarized in Table 1.

**Table 1:** Evidence layers relevant to airway hydration homeostasis in patients with artificial airways.

Evidence layer	Representative content	Suggested interpretation
Established clinical evidence	Need for humidification during invasive ventilation; approximate humidity targets; HH/HME indications and limitations	Supports current clinical practice and guideline-based recommendations.
Mechanistic and disease-model evidence	CFTR/ENaC regulation, PCL^a^ compression, mucin hyperconcentration, inflammatory changes in mucus solids	Explains plausible biological pathways, but should not be treated as the only proven mechanism in all patients with artificial airways.
Translational and engineering evidence	Ventilator waveform indices, artificial cilia sensors, microfluidic rheology, closed-loop humidification concepts	Represents early-stage approaches that require external validation and safety assessment.

a Periciliary layer: a brush-like structure formed by membrane-tethered mucins and macromolecules on the airway epithelial surface, which physically separates the overlying mucus layer from cilia to maintain normal mucociliary transport.

## 3 Physiological basis of airway hydration homeostasis

### 3.1 Thermal conditioning and the isothermic saturation boundary

During spontaneous breathing, inspired gas is progressively warmed and humidified by the nasal cavity, pharynx, and conducting airways. Under normal conditions, gas approaching the lower trachea is close to body temperature and saturated with water vapor, corresponding to an absolute humidity of approximately 44 mg/L at 37 °C (1–3). The region at which inspired gas reaches body temperature and full saturation is often referred to as the isothermic saturation boundary (ISB).

Artificial airway placement displaces this conditioning process distally. When delivered gas is insufficiently humidified, the lower airway must supply additional water and heat to the inspired gas. The resulting evaporative demand may reduce ASL volume, alter mucus concentration, and injure epithelial surfaces. Absolute humidity is more informative than relative humidity in this setting because it reflects the actual mass of water available per liter of inspired gas.

### 3.2 Airway surface liquid, the periciliary layer, and mucociliary clearance

ASL is commonly conceptualized as a mucus layer overlying a periciliary layer (PCL). More recent models describe the PCL as a brush-like structure formed by membrane-tethered mucins and other macromolecules that prevent the mucus layer from compressing cilia (4). Adequate PCL hydration preserves the space required for ciliary beating and coordinated mucus transport. When hydration is reduced or mucus osmotic pressure increases, cilia may become mechanically constrained, resulting in impaired MCC (4, 5).

MCC is a key airway defense mechanism. It depends not only on ciliary function but also on mucus properties. Secretions must be sufficiently cohesive to capture inhaled particles and pathogens, yet sufficiently mobile to be transported by cilia or removed by cough or suction. Artificial airways modify this balance by changing airflow, bypassing upper-airway conditioning, reducing cough effectiveness, and creating surfaces for secretion deposition.

### 3.3 Mucus biophysics and secretion clearability

Mucus rheology is determined primarily by gel-forming mucins, particularly MUC5AC and MUC5B, along with ions, proteins, extracellular DNA, actin, lipids, and inflammatory cell products (6, 7, 11). A moderate viscoelastic structure favors particle trapping and transport, whereas excessive solid concentration increases elasticity, adhesiveness, and yield stress. Mucus solids concentration has therefore been proposed as a biophysical marker of airway disease severity and secretion clearability (7).

In the context of artificial airways, the most clinically relevant endpoint is not whether mucus meets a specific molecular definition of dehydration, but whether it remains transportable, suctionable, and unlikely to form obstructive plugs. This pragmatic endpoint bridges molecular mechanisms and bedside humidification management.

## 4 Mechanisms of hydration imbalance after artificial airway placement

### 4.1 Evaporative water loss and epithelial injury

The most direct mechanism of airway dehydration is evaporative water loss caused by delivery of inadequately humidified gas to the lower respiratory tract. Fever, high minute ventilation, prolonged mechanical ventilation, circuit leaks, inappropriate humidifier settings, and interruptions in active humidification may increase water loss (1, 18). Experimental and clinical observations suggest that insufficient humidity can impair mucosal function and secretion handling (3).

Repeated or prolonged dehydration may damage the airway epithelium, alter epithelial barrier integrity, and increase susceptibility to mechanical irritation and infection. These processes are likely modulated by underlying lung disease, inflammatory state, ventilatory pattern, and the duration of artificial airway use.

Additionally, ambient environmental conditions—such as room temperature and relative humidity—play a critical role in humidification dynamics by influencing thermal loss and condensation within the breathing circuit (2, 29).

### 4.2 Epithelial ion transport and ASL regulation

ASL volume is heavily regulated by epithelial ion transport, notably CFTR-mediated anion secretion and ENaC-mediated sodium absorption. While these regulatory pathways are well established in chronic airway conditions (e.g., cystic fibrosis, asthma, and tobacco smoke exposure) (5, 8–10), their relevance to artificial airways must be interpreted with caution. In mechanically ventilated patients, dehydration is initiated predominantly by physical bypass of the upper airway and evaporative gas stress rather than primary channelopathies. Although secondary dysregulation of ion channels may exacerbate mucosal dryness during severe inflammatory states, it should not be regarded as a universal driver of airway dehydration in intubated patients.

### 4.3 Inflammation, infection, and biofilm-associated secretion burden

Inflammation alters mucus composition and rheology through biologically plausible mechanisms. Neutrophilic inflammation increases extracellular DNA, proteases, and cellular debris, which are associated with elevated mucus solids concentration and viscoelasticity in preclinical and observational studies (6, 7, 9). Concurrently, secretion retention is observationally linked to bacterial colonization and biofilm accumulation on endotracheal tube surfaces (13, 14).

However, while impaired hydration may contribute to secretion stagnation, airway dehydration should not be interpreted as an independent etiology of ventilator-associated pneumonia (VAP), which has a complex, multifactorial pathogenesis. Rather, the interplay among hydration, infection, and inflammation represents a biologically plausible feedback framework in which reduced hydration facilitates retention and secondary inflammation further alters secretion biophysics.

### 4.4 Biophysical progression from concentrated mucus to secretion obstruction

Polymer physics models suggest that severe water loss and hyperconcentration of mucin networks can drive mucus into a glassy or highly elastic state. In clinical context, this “glass-like transition” serves as a useful biophysical analogy to describe extreme loss of clearability rather than a proven, irreversible state occurring in every intubated patient (7, 11). Clinically, secretions move along a fluid-to-solid spectrum, and whether dehydration causes life-threatening luminal obstruction depends on humidification adequacy, secretion burden, baseline airway caliber, and suctioning performance.

**Figure 1:**
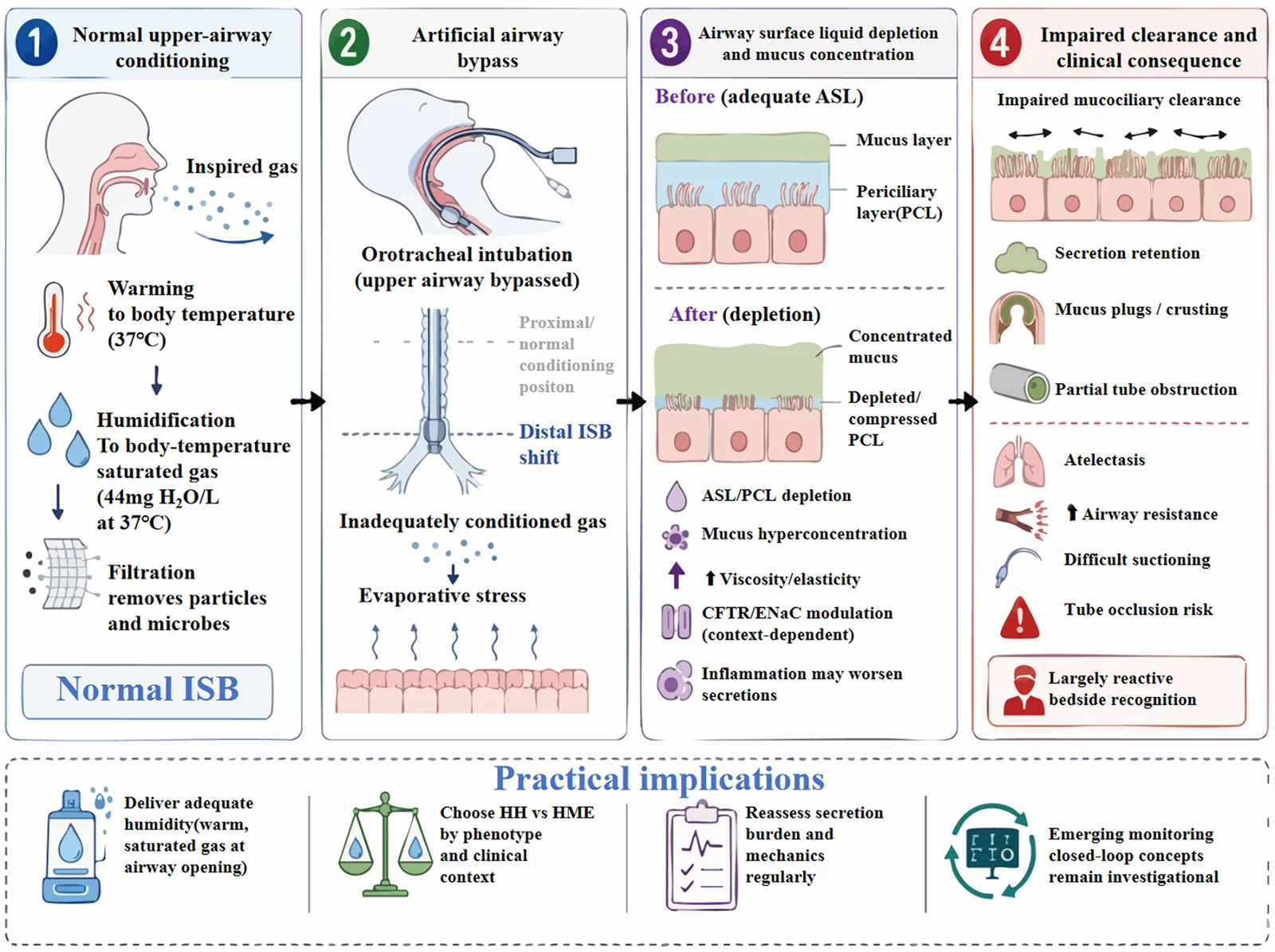
Conceptual pathway from upper-airway bypass to impaired airway hydration and secretion-related complications. Under physiological conditions, the upper airway warms, humidifies, and filters inspired gas before the isothermic saturation boundary is reached. Orotracheal intubation bypasses these functions and shifts the conditioning requirement distally. When delivered gas is inadequately conditioned, evaporative stress may reduce airway surface liquid and periciliary layer hydration, promote mucus hyperconcentration and increased viscoelasticity, and impair mucociliary clearance. These changes may contribute to secretion retention, mucus plugs or crusting, partial endotracheal tube obstruction, atelectasis, increased airway resistance, difficult suctioning, and tube occlusion. CFTR/ENaC modulation and inflammatory effects are shown as context-dependent mechanistic contributors rather than universally established pathways in mechanically ventilated patients. The lower panel summarizes practical implications, including adequate humidity delivery, individualized HH/HME selection, repeated reassessment, and the investigational status of emerging monitoring and closed-loop approaches. The schematic is conceptual and is not drawn to anatomical scale. ASL, airway surface liquid; PCL, periciliary layer; ISB, isothermic saturation boundary; MCC, mucociliary clearance; ETT, endotracheal tube; HH, heated humidifier; HME, heat-and-moisture exchanger; CFTR, cystic fibrosis transmembrane conductance regulator; ENaC, epithelial sodium channel.

Figure 1 provides a conceptual synthesis of the sequence from loss of upper-airway conditioning to distal displacement of the isothermic saturation boundary, evaporative stress, altered mucus biophysics, impaired clearance, and secretion-related clinical consequences.

## 5 Bedside assessment and clinical consequences

Current bedside evaluation of airway hydration remains largely reactive. In routine clinical practice, early changes in secretion characteristics or respiratory mechanics may precede overt secretion-related complications, but these findings are non-specific and must be interpreted in the overall clinical context. For practical bedside assessment, findings can be organized into three levels: secretion-related warning signs, respiratory-mechanics or ventilator clues, and established clinical consequences of impaired secretion clearance.

### 5.1 Secretion-related early warning signs

The earliest bedside clues are changes in secretion characteristics. Thick, tenacious, or sticky secretions, altered secretion appearance, increasing difficulty mobilizing secretions, and difficulty passing a suction catheter may suggest inadequate humidification or reduced secretion clearability. These findings are not specific for airway dehydration and may also be influenced by infection, inflammation, underlying lung disease, and secretion production.

Suctioning frequency alone should not be regarded as a surrogate marker of airway hydration because it is influenced by secretion burden, cough effectiveness, sedation, endotracheal-tube characteristics, staffing practices, and local suctioning protocols. Routine or unnecessary suctioning may itself cause mucosal trauma, bleeding, derecruitment, hypoxemia, and patient discomfort. Bedside assessment should therefore integrate secretion characteristics with respiratory mechanics and the overall clinical context rather than rely on suctioning frequency alone (20).

### 5.2 Respiratory mechanics and ventilator waveform clues

Ventilator waveforms provide continuous non-invasive information about the patient-ventilator system. Secretion retention may produce irregular expiratory flow oscillations, changes in airway resistance, prolonged expiratory time constants, or altered pressure-flow relationships. These signs are not specific to airway hydration imbalance and may also reflect bronchospasm, circuit water, air leak, patient effort, or asynchrony.

At the bedside, progressive increases in peak airway pressure or in the peak-to-plateau pressure difference may suggest an increasing resistive load when lung compliance remains relatively stable. Abnormal expiratory flow patterns or improvement in airway resistance after suctioning may further support a secretion-related component. However, none of these findings is specific for inadequate humidification, and alternative causes such as bronchospasm, tube kinking, circuit condensation, air leak, or patient-ventilator asynchrony must be considered.

Recent modeling work has explored waveform-derived secretion indices for estimating hidden secretion burden during mechanical ventilation (21). Such approaches are attractive because they use data already generated by ventilators, but they require validation across ventilator modes, lung mechanics phenotypes, and clinical settings before they can guide humidification adjustment.

### 5.3 Established clinical consequences of impaired secretion clearance

When secretion retention progresses, more overt clinical consequences may develop. These include visible airway or endotracheal-tube crusting, mucus plugging, partial or complete tube obstruction, increased airway resistance, impaired ventilation or oxygenation, and atelectasis. In severe cases, secretion retention may necessitate bronchoscopic clearance and may contribute to difficulty during ventilator weaning. These findings should be interpreted as consequences of impaired secretion clearance rather than as specific markers of airway dehydration.

### 5.4 Imaging, bronchoscopy, and rheological assays

Chest imaging and lung ultrasound can identify downstream consequences such as atelectasis or consolidation and may provide indirect evidence of secretion retention, but they do not directly measure airway or mucus hydration (30, 31). While bronchoscopy enables direct visualization and therapeutic clearance of obstructive mucus plugs, it should be regarded as an invasive diagnostic and interventional tool for specific clinical indications rather than a reference standard for evaluating airway hydration status, unless integrated into a validated clinical scoring system. Laboratory rheological assays, including magnetic wire microrheology, can quantify viscosity, elastic modulus, relaxation behavior, and mucus heterogeneity (12).

For clinical translation, rheological measures must be linked to actionable thresholds. A practical research priority is to determine whether specific rheological or waveform-derived metrics predict mucus plugging, suctioning burden, VAP risk, ventilator duration, or failure to wean.

## 6 Current humidification strategies

### 6.1 Target physical parameters: humidity, temperature, and monitoring sites

Current AARC guidance recommends that active humidification during invasive mechanical ventilation deliver an absolute humidity of 33–44 mg H_2_O/L, with a gas temperature of 34–41 °C and 100% relative humidity at the circuit Y-piece (2). When passive humidification is used during invasive ventilation, an HME should provide at least 30 mg H_2_O/L (2). During non-invasive ventilation (NIV), active humidification may improve comfort and tolerance; however, a universally validated absolute-humidity threshold for NIV has not been established. Key quantitative targets and operational considerations are summarized in Table 2.

**Table 2:** Quantitative targets and operational considerations for humidification during respiratory support.

Modality or parameter	Evidence-supported value or condition	Clinical interpretation	Limitations and context
Physiological reference condition	Fully saturated gas at 37 °C contains approximately 44 mg H_2_O/L (1–3).	Provides a physiological reference for gas conditioning.	This physiological reference should not be interpreted as a mandatory minimum clinical target for every device or patient.
Active humidification during invasive mechanical ventilation	AARC guidance: absolute humidity 33–44 mg H_2_O/L, gas temperature 34–41 °C, and 100% relative humidity at the circuit Y-piece (2).	Represents the principal guideline-supported operating range for active humidification during invasive ventilation.	Device settings alone do not guarantee delivered humidity; circuit configuration, probe position, ambient temperature, chamber water, and heater-wire performance can alter output.
Passive humidification with an HME	AARC guidance: HME moisture output should be at least 30 mg H_2_O/L during invasive mechanical ventilation (2).	Provides a minimum performance criterion when passive humidification is selected.	The value is a device-performance criterion rather than a patient-specific hydration threshold; actual output is device- and condition-dependent.
HME dead space and low tidal volume	HMEs add device-specific instrumental dead space and airflow resistance; their effects become more relevant when tidal volume is small (2).	Consider an HH when added dead space or resistance becomes clinically important.	No universal dead-space or resistance cutoff applies to all patients; clinical impact depends on device design, patient size, tidal volume, and respiratory mechanics.
Minute ventilation and circuit leak	High ventilatory demand and substantial leak can reduce the efficiency of passive humidification (1, 2). No universally validated cutoff has been established.	Reassess HME suitability when ventilatory demand or leak becomes substantial.	Effects are device- and context-dependent; study-specific minute-ventilation or leak values should not be converted into universal thresholds.
HME inspection and replacement	HMEs should be inspected regularly and replaced earlier if visibly contaminated, saturated, or obstructed (1, 2).	Early replacement is appropriate when secretion accumulation increases resistance or impairs device function.	Replacement intervals remain device- and manufacturer-specific; no universal interval is established for all patients and HME designs.
HH circuit and environmental factors	Delivered humidity is influenced by ambient and inlet-gas temperature, heated-wire function, chamber water, circuit configuration, and temperature-probe position (34).	Check system factors when under-humidification or excessive condensation is suspected.	A set temperature does not guarantee a specific delivered absolute humidity; humidifier generations and control algorithms may behave differently.
Interruption of active humidification	Bench data showed absolute humidity falling to <30 mg H_2_O/L within minutes, about 15 mg H_2_O/L by 15–20 min, and <10 mg H_2_O/L by 1 h after HH shutdown; >30 mg H_2_O/L returned within about 5 min after restart (18).	Active humidification should be restored promptly after transport, procedures, or circuit interruptions.	These are bench hygrometric data, not validated clinical injury thresholds or evidence of a “safe” interruption duration.
NIV/HFOT and active humidification	AARC guidance supports active humidification during NIV to improve adherence and comfort; no universally validated absolute-humidity threshold for initiation has been established (2, 33).	Consider active humidification when dryness, poor tolerance, high flow or leak, or difficult secretion clearance occurs.	Because the upper airway remains anatomically present, the goals differ from humidification after complete upper-airway bypass.
HH versus HME: clinical outcomes	A Cochrane review found no overall difference in airway blockage, pneumonia, or mortality between HME and HH strategies, with substantial heterogeneity across studies (15).	Supports individualized device selection rather than universal preference for one modality.	Absence of overall superiority should not be interpreted as therapeutic equivalence in every patient phenotype or clinical scenario.

AARC, American Association for Respiratory Care; AH, absolute humidity; HFOT, high-flow oxygen therapy; HH, heated humidifier; HME, heat-and-moisture exchanger; NIV, non-invasive ventilation; RH, relative humidity; VT, tidal volume.

Numerical values represent guideline recommendations or findings reported under specified experimental conditions. Device-specific measurements and study-specific thresholds should not be interpreted as universal physiological cutoffs unless clinically validated.

Temperature sensor positioning is critically determinant of true humidity delivery: probes located at the humidifier chamber exit regulate heating energy based on saturated vapor temperature, whereas distal airway probes (near the Y-piece) monitor gas cooling along the circuit (34). Misplacement or thermal interference (e.g., incubation under warming blankets or exposure to direct air conditioning airflow) distorts feedback loops, inducing either under-humidification or excessive precipitation (34).

These patient-level clinical targets should be distinguished from equipment-level performance requirements. ISO 80601-2-74:2026 specifies basic safety and essential performance requirements for respiratory humidifying equipment and associated accessories (32). Such equipment-level requirements should not be interpreted as patient-specific physiological humidification thresholds.

### 6.2 Heated humidifiers

Heated humidifiers actively add heat and water vapor to inspired gas and are generally recommended whenever prolonged invasive mechanical ventilation is anticipated or when passive humidification may be insufficient (1, 2). According to current guidelines, HHs are preferred in patients with copious or thick airway secretions, high minute ventilation, tracheostomy, hypothermia, or contraindications to heat-and-moisture exchangers (HMEs) (1, 2). Active humidification mitigates excessive respiratory heat loss and hypothermia risk for long-term mechanically ventilated critical care patients; for patients receiving tracheostomy, it also improves mucus clearance relative to cold-air nebulization (16, 17).

Under appropriate operating conditions, modern heated humidifiers are capable of delivering gas close to physiological conditions (approximately 44 mg H_2_O/L at 37 °C). However, actual humidity delivery depends on multiple technical and environmental factors rather than device settings alone. These include inspiratory flow, circuit length and configuration, ambient temperature, water chamber volume, heater wire performance, and the location of temperature probes (34). Cooling of inspired gas within the circuit promotes condensate formation, particularly when circuit temperature falls below the dew point. Excessive condensate not only reduces effective humidity delivery but may also increase contamination risk, interfere with ventilator triggering, or require circuit manipulation that interrupts ventilation.

Importantly, use of an HH does not by itself guarantee adequate gas conditioning. High ambient temperature or elevated ventilator outlet gas temperature can reduce the humidity delivered by heated-wire humidifiers under certain operating conditions (34). In a retrospective study of mechanically ventilated patients with COVID-19, three endotracheal-tube occlusions occurred among 14 patients managed in an ICU using heated-wire HHs; after measures were implemented to reduce under-humidification, including heater-plate temperature monitoring, no further occlusions were observed (35). Other COVID-19 series have described ETT obstruction associated with thick, tenacious mucin, cellular debris, and tracheobronchial slough (36, 37). These findings suggest that both humidifier operating conditions and disease-related secretion burden should be considered when evaluating the risk of ETT obstruction.

Clinical management should therefore include regular inspection of water chamber filling, appropriate positioning of temperature probes, standardized handling of condensate, and maintenance of circuit integrity. Interruptions of active humidification during patient transport, imaging procedures, or surgery may transiently expose the airway to inadequately conditioned gas, particularly during prolonged interruptions. However, evidence remains insufficient to define a validated duration beyond which interruption becomes clinically harmful, and no evidence-based threshold is currently available.

### 6.3 Heat-and-moisture exchangers

Heat-and-moisture exchangers provide passive humidification by conserving heat and water from exhaled gas and returning them during inspiration. Their simplicity, portability, low maintenance requirements, and reduced circuit condensation make them suitable for short-term mechanical ventilation, anesthesia, patient transport, and carefully selected stable patients with relatively low secretion burden (1, 2, 15).

Unlike heated humidifiers, HMEs do not generate additional water vapor, and their performance depends on the patient's own exhaled heat and moisture. Consequently, several clinical conditions may reduce HME efficiency, including high minute ventilation, substantial air leak, hypothermia, copious or thick secretions, low tidal volume ventilation, pediatric airways, and tracheostomy (1, 2). HMEs introduce device-specific instrumental dead space and airflow resistance, the clinical importance of which depends on device design, tidal volume, patient size, and respiratory mechanics. However, no universally accepted numerical threshold exists for defining clinically significant dead space or resistance because these effects depend on patient size, ventilatory settings, and respiratory mechanics.

HME saturation or obstruction represents another important limitation. Progressive accumulation of secretions may increase resistance, impair humidification efficiency, and occasionally result in complete airway obstruction, necessitating immediate device replacement. For this reason, HMEs require regular inspection and replacement according to manufacturer recommendations or earlier if visibly contaminated or obstructed.

A Cochrane systematic review comparing HMEs and heated humidifiers found no consistent superiority of either strategy regarding mortality, ventilator-associated pneumonia, airway occlusion, duration of mechanical ventilation, or intensive care unit length of stay (15). However, substantial clinical heterogeneity among included studies—including differences in patient populations, ventilator settings, device characteristics, and outcome definitions—limited the certainty of evidence. Therefore, the absence of overall superiority should not be interpreted as therapeutic equivalence across all patient populations.

### 6.4 Special clinical scenarios

Unlike invasive artificial airways (endotracheal intubation and tracheostomy) that completely bypass the anatomical upper airway, non-invasive support modalities such as NIV and HFOT preserve upper airway structures. However, high inspiratory gas flow rates, mouth breathing, and circuit leaks can still impose marked evaporative stress on the nasal and oral mucosa. Therefore, while humidification in invasive artificial airways aims primarily to substitute for bypassed upper airway thermal conditioning and prevent epithelial injury, humidification during non-invasive support focuses principally on optimizing patient comfort, interface tolerance, and secretion mobility (1, 19).

Pediatric patients and patients with tracheostomy require particular attention. Small airway diameter means that a modest amount of dried secretion can substantially increase resistance. In tracheostomy, the bypassed upper airway and reduced ability to condition inspired gas make continuous humidification especially important.

**Figure 2:**
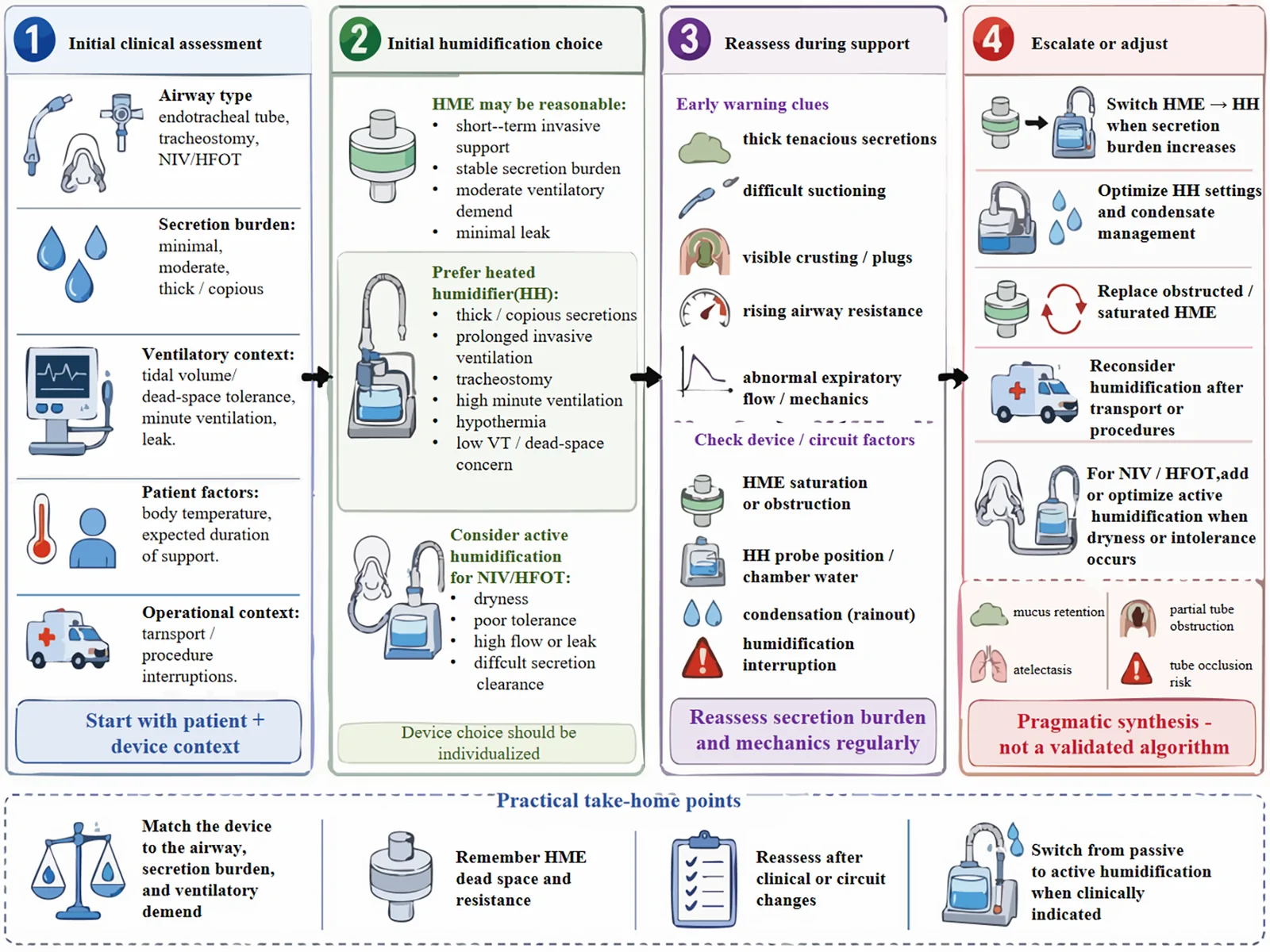
Pragmatic framework for initial humidification-device selection and dynamic reassessment during respiratory support. Initial assessment integrates airway type, secretion burden, tidal-volume and dead-space considerations, minute ventilation, leak, body temperature, anticipated duration of support, and potential interruptions during transport or procedures. HME use may be reasonable for selected patients requiring short-term invasive support with stable secretions, moderate ventilatory demand, and minimal leak. An HH is generally favored when secretions are thick or copious, invasive support is prolonged, tracheostomy is present, minute ventilation is high, hypothermia is present, or HME-related dead space is a concern. During NIV or HFOT, active humidification may be considered when dryness, poor tolerance, high flow or leak, or difficult secretion clearance occurs. Reassessment should include secretion characteristics, suctioning difficulty, respiratory mechanics, HME saturation or obstruction, HH probe and chamber status, condensate, and interruptions in humidification. Corrective actions may include switching from an HME to an HH, optimizing HH settings and condensate management, replacing an obstructed or saturated HME, and reassessing humidification after transport or procedures. This framework represents a practical synthesis of the evidence reviewed and should not be interpreted as a prospectively validated clinical decision algorithm. The categories shown are contextual and do not represent universal numerical thresholds. HME, heat-and-moisture exchanger; HH, heated humidifier; NIV, non-invasive ventilation; HFOT, high-flow oxygen therapy; VT, tidal volume.

Based on the evidence summarized above, Figure 2 presents a pragmatic framework for initial humidification-device selection and repeated reassessment during respiratory support. It is intended as a clinical synthesis rather than a validated decision algorithm.

## 7 Emerging monitoring and closed-loop management technologies

Emerging approaches relevant to airway hydration assessment and humidification management span markedly different levels of technological and clinical maturity. Some approaches directly assess airway secretions or secretion-related respiratory mechanics, whereas others represent broader engineering concepts that may inform future airway applications. To avoid treating these technologies as equivalent, this section organizes them into four descriptive developmental categories: computational signal analysis, *ex vivo* secretion characterization, proof-of-concept *in situ* sensing, and conceptual closed-loop humidification. The potential value and current limitations of these approaches are summarized in Table 3.

**Table 3:** Monitoring approaches and translational technologies for airway hydration assessment.

Technology	Potential value	Current limitation
Ventilator waveform indices	Continuous, non-invasive screening for secretion accumulation and secretion-related mechanics changes	Not a direct measure of hydration; limited specificity and external validation across ventilator modes and patient phenotypes.
Microrheological assays	Quantitative characterization of mucus viscosity, elasticity, and rheological heterogeneity	*Ex vivo* sampling, handling effects, limited bedside availability, and absence of validated actionable thresholds.
Artificial cilia or flexible airway sensors	Proof-of-concept in situ assessment of mucus viscosity and fluid-layer thickness	Evidence largely limited to phantom/*ex vivo* models; applicability to ETTs and prolonged ventilation remains untested; fouling and retrieval concerns.
Closed-loop humidification frameworks	Potential integration of physiological and device signals to individualize humidification delivery	Conceptual; no established airway-hydration control system; requires validated sensors, safety limits, human override, and staged clinical evaluation.

### 7.1 Tier 1: computational signal analysis and clinical decision support

This tier represents the approach closest to current clinical translation, with the potential to provide non-invasive and continuous estimation of secretion accumulation and secretion-related changes in respiratory mechanics without interrupting mechanical ventilation. Importantly, these waveform-derived indices should not currently be interpreted as direct measurements of airway or mucus hydration. Candidate indices can be derived from routinely available ventilator signals and features, including airway pressure, flow, tidal volume, inspiratory flow characteristics, and expiratory time constants (21). Future validation should compare waveform-derived indices with clinically meaningful comparators, such as prospectively assessed secretion burden, suctioned secretion quantity or properties, changes in respiratory mechanics after airway clearance, and bronchoscopy findings when bronchoscopy is clinically indicated. Current evidence is exemplified by physiology-informed modeling combined with retrospective or clinically collected ventilator waveform data, supporting an early clinical-evaluation stage rather than established bedside implementation (21). A major implementation concern is limited diagnostic specificity. Waveform abnormalities associated with secretion accumulation may overlap with changes caused by bronchospasm, circuit condensation, endotracheal-tube biting or kinking, air leaks, patient effort, or patient–ventilator asynchrony. Accordingly, waveform-derived secretion indices would need to be interpreted together with the clinical and ventilator context rather than used as stand-alone indicators of inadequate humidification. Before routine clinical use, waveform-derived indices require prospective external validation across different ventilator modes, secretion burdens, and respiratory-mechanics phenotypes. Studies should first establish diagnostic performance, reproducibility, and incremental value over conventional bedside assessment before evaluating whether waveform-guided management improves patient-centered clinical outcomes.

### 7.2 Tier 2: *ex vivo* secretion characterization and rheological phenotyping

This tier focuses on direct quantitative characterization of airway secretion properties, particularly viscosity, elasticity, relaxation behavior, and rheological heterogeneity. These measurements may support biophysical secretion phenotyping, but their role in guiding humidification or mucoactive therapy remains investigational. Microrheological methods can operate on relatively small airway-secretion samples, which may be advantageous when sample availability is limited (12). Depending on the platform, methodological validation may include comparison with conventional rheometry, gravimetric solids measurements, or other established physical characterization methods. Available evidence remains predominantly *ex vivo* or laboratory based, including measurements of human respiratory mucus and experimental microfluidic or rheological systems (12, 23).

Key barriers to bedside translation include sample collection and handling, changes in secretion properties during storage or processing, equipment availability, turnaround time, inter-sample heterogeneity, and the absence of validated clinically actionable thresholds. Future development should prioritize standardized sampling, reproducible point-of-care measurements, rapid turnaround, and prospective studies linking rheological measures to clinically relevant outcomes and treatment responses.

### 7.3 Tier 3: proof-of-concept *in situ* biosensors and implantable electronics

In situ sensing offers the possibility of directly characterizing secretion properties within the airway, but current evidence remains largely proof-of-concept. A representative example is the sensory artificial-cilia platform described by Wang et al., which used magnetically actuated flexible sensing elements to estimate mucus viscosity and fluid-layer thickness (22). The system was evaluated in airway phantoms and *ex vivo* sheep trachea, including configurations integrated with airway stents (22). These findings do not yet establish applicability to endotracheal tubes, prolonged mechanical ventilation, or humidification control in ICU patients.

More broadly, advances in physically transient electronics may offer engineering concepts for temporary biomedical sensors that avoid permanent device retention (27). Similarly, advances in implantable radiofrequency communication may provide general design principles for future wireless airway sensors (28). However, neither technology has yet been validated specifically for airway hydration monitoring or integration with artificial airways, and their relevance in this context remains prospective.

Major implementation challenges include biofouling, mucus encrustation, biofilm formation, signal drift, degradation in high-humidity environments, interference with suctioning, mucosal injury, and device dislodgement or retention. Before clinical translation, airway-mounted sensors would require biocompatibility evaluation in accordance with relevant standards, assessment of anti-fouling performance, long-term signal stability, compatibility with routine airway care, and safe retrieval or degradation strategies.

### 7.4 Tier 4: integrated closed-loop humidification frameworks

As the most integrated and technologically complex, but least clinically mature approach, closed-loop humidification should currently be regarded as a conceptual framework rather than an established clinical technology. A future system would aim to adjust humidifier output dynamically in response to continuously updated physiological and device-related information (25, 26).

Digital-twin approaches developed more broadly in critical care provide a conceptual framework for integrating heterogeneous physiological data into patient-specific computational models (24). Applied hypothetically to airway humidification, such a framework could combine thermodynamic, ventilatory, and secretion-related information. Whether this approach can reliably estimate regional airway hydration or improve humidification management has not yet been established.

A hypothetical closed-loop humidification system could integrate delivered gas temperature and absolute humidity, minute ventilation, ventilator waveforms, body temperature, circuit or interface leak, and selected indicators of secretion burden. Future validation would need to compare automated control with guideline-based clinician management and predefined humidity and temperature safety targets. Because such integrated airway-humidification systems have not yet undergone direct clinical validation, this tier should be regarded as conceptual.

Potential safety concerns include control-loop instability, sensor failure or drift, inappropriate under- or over-humidification, excessive circuit condensation, and automation bias. Any future system would require predefined hardware and software safety limits, alarms, manual override, failure-mode analysis, and staged evaluation through benchtop testing, relevant preclinical models, first-in-human feasibility studies, and, if justified, subsequent controlled clinical trials.

## 8 Research gaps and future directions

Several gaps should guide future research. First, mechanistic studies of ASL dehydration need to be linked more directly to artificial-airway populations. Many current concepts originate from cystic fibrosis, asthma, smoking exposure, or *in vitro* mucus studies. Prospective studies in mechanically ventilated patients are needed to determine which mechanisms dominate in different clinical phenotypes.

Second, standardized hydration endpoints are lacking. Future trials should consider absolute humidity target attainment, secretion rheology, suctioning burden, mucus plugging, atelectasis, VAP, duration of mechanical ventilation, ICU length of stay, comfort, and device-related adverse events. Linking biophysical markers to clinical endpoints will be essential.

Third, special populations remain underrepresented. Patients with tracheostomy, pediatric airways, high-flow therapy, severe ARDS, COPD exacerbation, sepsis, and high secretion burden may have distinct humidification needs. A single fixed parameter is unlikely to match all patients.

Fourth, translational technologies require staged validation. *In situ* sensors and closed-loop control systems should be evaluated for biocompatibility, anti-fouling performance, signal stability, electromagnetic interference resistance, infection risk, human factors, and regulatory feasibility before clinical outcome trials are attempted.

## 9 Conclusions

Invasive artificial airways bypass the natural conditioning functions of the upper respiratory tract, rendering appropriate artificial humidification an essential component of mechanical ventilation. Current clinical management relies on heated humidifiers (HHs) and heat-and-moisture exchangers (HMEs). Because these devices possess distinct biophysical constraints, device selection must be individualized based on secretion burden, ventilation strategy, minute ventilation, circuit leaks, patient temperature, and anticipated duration of support. At present, bedside assessment of airway hydration remains largely reactive, depending on delayed physical indicators such as thick secretions, rising airway resistance, or tube encrustation rather than proactive monitoring.

Understanding airway hydration mechanisms requires recognizing current limitations in the evidence base. Many proposed cellular and biophysical pathways—including mucus concentration, epithelial evaporative stress, and impaired mucociliary transport—are derived primarily from experimental benchtop models or chronic respiratory diseases. While these models offer fundamental physiological insights, clinicians must exercise caution when extrapolating these mechanistic findings directly to acute critically ill patients receiving mechanical ventilation.

Emerging technological approaches, including computational ventilator waveform analytics, *ex vivo* mucus rheology, localized biosensors, and automated closed-loop humidification systems, represent innovative research avenues. However, these tools remain strictly in experimental, conceptual, or early translational stages and are not currently established clinical practices. Clinical humidification management should firmly adhere to evidence-based guideline recommendations while maintaining a clear distinction between proven interventions and future engineering concepts. Future progress will depend on rigorous clinical validation to determine whether advancing beyond reactive bedside assessments can ultimately improve patient-centered outcomes.
