# Supplementary material for: Airway hydration homeostasis in artificial airways: from mucus biophysics to monitoring and humidification strategies in critical care: a narrative review
Source: Front Med (Lausanne). 2026 Sep 11;13:1922639. doi: 10.3389/fmed.2026.1922639 (PMC13613053; doi:10.3389/fmed.2026.1922639)
Supplement: Supplementary file 1 [file Table1.docx]

**Supplementary Table S1. Literature search strategy and study selection procedures.**

| **Domain** | **Item** | **Details** |
| --- | --- | --- |
| Search framework | Review type | Narrative review with a structured, non-systematic literature search. |
| Search framework | Information sources | PubMed; Web of Science Core Collection; and major clinical guideline repositories. |
| Search framework | Last search date and language | Searches were updated through May 2026. Only English-language publications were included. |
| Search framework | Search fields | PubMed: Medical Subject Headings (MeSH) and Title/Abstract fields. Web of Science Core Collection: Topic field (TS), including title, abstract, author keywords, and Keywords Plus. |
| Search framework | Core concept blocks | Artificial airway: "artificial airway", "endotracheal intubation", and "tracheostomy".  Hydration/humidification: "airway humidification", "airway hydration", and "airway surface liquid".  Mucus and clearance: "mucus biophysics", "mucus rheology", and "mucociliary clearance".  Devices: "heated humidifier" and "heat and moisture exchanger". |
| Database-specific strategy | PubMed search string | (("artificial airway"[Title/Abstract] OR "artificial airways"[Title/Abstract] OR "endotracheal intubation"[Title/Abstract] OR "Intubation, Intratracheal"[MeSH Terms] OR tracheostom*[Title/Abstract] OR "Tracheostomy"[MeSH Terms]) AND  ("airway humidification"[Title/Abstract] OR "airway hydration"[Title/Abstract] OR "airway surface liquid"[Title/Abstract] OR humidif*[Title/Abstract] OR "Humidifiers"[MeSH Terms]) AND  ("mucus biophysics"[Title/Abstract] OR "mucus rheology"[Title/Abstract] OR "mucociliary clearance"[Title/Abstract] OR "Mucociliary Clearance"[MeSH Terms]) AND  ("heated humidifier"[Title/Abstract] OR "heated humidifiers"[Title/Abstract] OR "heat and moisture exchanger"[Title/Abstract] OR "heat-and-moisture exchanger"[Title/Abstract] OR HME[Title/Abstract] OR HMEs[Title/Abstract])) |
| Database-specific strategy | Web of Science Core Collection search string | TS=(("artificial airway*" OR "endotracheal intubation" OR tracheostom*) AND  ("airway humidification" OR "airway hydration" OR "airway surface liquid" OR humidif*) AND  ("mucus biophysics" OR "mucus rheology" OR "mucociliary clearance") AND  ("heated humidifier*" OR "heat and moisture exchanger*" OR "heat-and-moisture exchanger*" OR HME OR HMEs)) |
| Database-specific strategy | Clinical guideline repositories | The core concepts were adapted for targeted searches of major clinical guideline repositories through May 2026. |
| Eligibility and evidence framing | Eligible publication types | Clinical practice guidelines; randomized controlled trials; observational and physiological studies; biological or chronic-airway disease models relevant to airway surface liquid, mucus, or mucociliary clearance; and engineering or translational studies and prototypes relevant to airway monitoring or humidification. |
| Eligibility and evidence framing | Relevance and exclusion criteria | Records were retained when title and abstract screening indicated relevance to physiological, physical, technical, or clinical aspects of airway hydration. Duplicate records, non-English publications, and records outside the review scope were excluded. |
| Eligibility and evidence framing | Evidence interpretation | Direct clinical evidence was distinguished from mechanistic evidence derived mainly from chronic airway disease or experimental models and from early-stage translational or engineering evidence. |
| Screening and appraisal | Deduplication | Duplicate records retrieved from PubMed and Web of Science Core Collection were removed before screening. |
| Screening and appraisal | Screening and study selection | Haishao Chen, Xunbin Qiu, and Kaisheng Ye jointly performed literature screening and study selection. |
| Screening and appraisal | Resolution of disagreements | Disagreements regarding study inclusion were adjudicated by the corresponding author, Qun Wang. |
| Screening and appraisal | Formal appraisal and reporting | No formal risk-of-bias assessment was performed, and no PRISMA flow diagram was prepared, because the work was designed as a narrative rather than a systematic review. |

**Abbreviations:** HME, heat-and-moisture exchanger; MeSH, Medical Subject Headings; PRISMA, Preferred Reporting Items for Systematic Reviews and Meta-Analyses; TS, Topic; WoS, Web of Science.

**Note:** Search syntax was adapted to each database. Formal risk-of-bias assessment and PRISMA flow-chart reporting were not undertaken because this was a narrative review.
